# Supplementary material for: SIRT3 Deficiency Enhances Ferroptosis and Promotes Cardiac Fibrosis via p53 Acetylation
Source: Cells. 2023 May 19;12(10):1428. doi: 10.3390/cells12101428 (PMC10217433; doi:10.3390/cells12101428)
Supplement: Supplementary file 1 [file cells-12-01428-s001.zip › cells-2366397-supplementary.pdf]

cells

MDPI

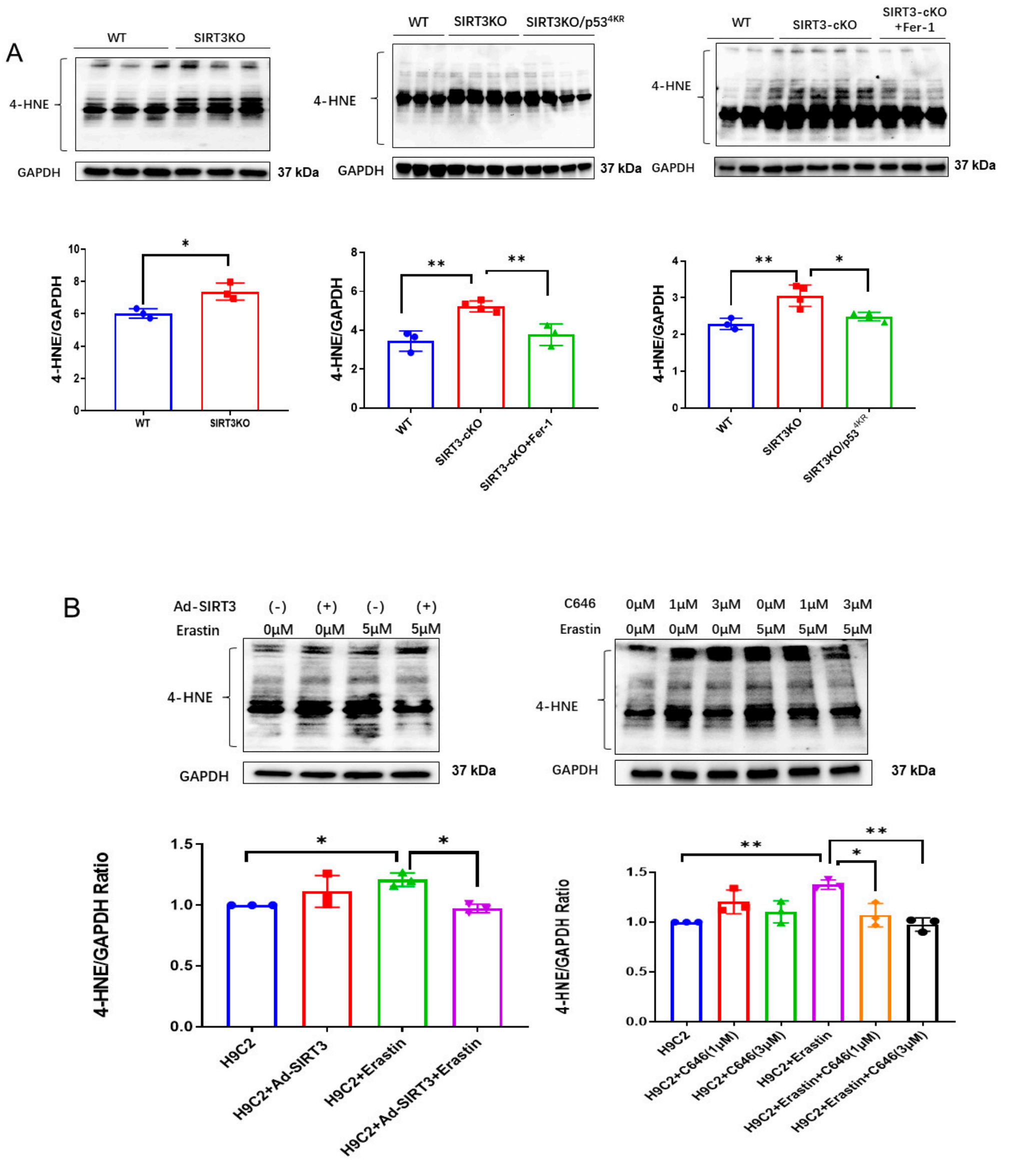

Supplemental Figure S1. Level of 4-HNE in indicated groups A) Representative immunoblots and analysis of 4-HNE and corresponding GAPDH in the indicated mouse hearts (n=3-4). **B)** Representative immunoblots and relative ratios of 4-HNE/GAPDH to the control H9c2 group in the indicated cells (n=3-4). Mean ± S.D., $^{**}p<0.01$, $^{*}p<0.05$.

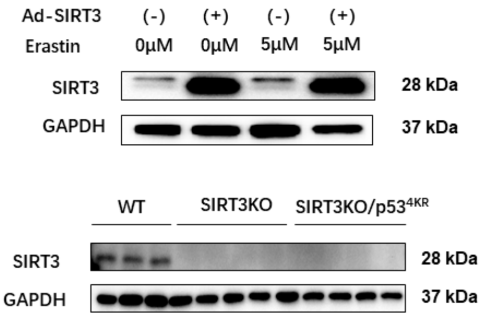

Supplemental Figure S2. Levels of SIRT3 in indicated groups. Representative immunoblots and levels of SIRT3 expression in the indicated H9c2 cell lines and mouse hearts. Ad-SIRT3 treatment increased levels of SIRT3 expression in H9c2 cell line with/out Erastin (Top). The expression of SIRT3 was absence in the hearts of SIRT3 KO mice and SIRT3KO/p53^{4KR} mice (Bottom).
